# Accurate Object Pose Estimation Using Depth Only

**DOI:** 10.3390/s18041045

**Published:** 2018-03-30

**Authors:** Mingyu Li, Koichi Hashimoto

**Affiliations:** Graduate School of Information Sciences, Tohoku University, Aramaki Aza Aoba 6-6-01, Aoba-Ku, Sendai 980-8579, Japan; koichi@m.tohoku.ac.jp

**Keywords:** pose estimation, point pair feature, point cloud

## Abstract

Object recognition and pose estimation is an important task in computer vision. A pose estimation algorithm using only depth information is proposed in this paper. Foreground and background points are distinguished based on their relative positions with boundaries. Model templates are selected using synthetic scenes to make up for the point pair feature algorithm. An accurate and fast pose verification method is introduced to select result poses from thousands of poses. Our algorithm is evaluated against a large number of scenes and proved to be more accurate than algorithms using both color information and depth information.

## 1. Introduction

Vision-based object recognition and pose estimation has been widely researched because of its importance in robotics applications. Given the CAD model of the object, the task is to recognize the object and estimate the 6 Degree-of-Freedom (DOF) pose accurately. Though a lot of works have been conducted, it is still a challenging task in computer vision because of sensor noise, occlusion and background clutter. Generally, the objects are captured by 2D/3D sensors and based on the vision sensors, three kinds of information are utilized for recognition: RGB, depth and RGB-D.

In order to estimate pose of objects using the RGB cameras, some research has been carried out. In [1], an approach for building metric 3D models of objects using local descriptors from several images was proposed. Given an input image, local descriptors are matched to the stored models online, using a novel combination of the RANSAC and Mean Shift algorithms to register multiple instances of each object. However, this method can only be used for the objects with texture in household environments. For the texture-less objects, Munoz et al. [2] proposed a method using the edge information with only one image as the input. The pose is estimated using edge correspondences, where the similarity measurement is encoded using a pre-computed linear regression matrix. However, the edge detection is heavily affected by the illumination conditions so that some research using MFC (Multi Flash Camera) [3,4,5] has been conducted. In [3], the silhouettes are segmented into different objects and each silhouette is matched across a database of object silhouettes in different poses to find the coarse pose. Liu et al. [4] proposed the Chamfer Matching method to extract the depth edge and the method is able to perform pose estimation within one second in an extremely cluttered environment. In [5], a method for finding a needle in a specular haystack is proposed by reconstructing the screw axis as a 3D line.

As 3D sensors are becoming more and more affordable, methods using point clouds or depth images are proposed [6,7,8,9,10]. Rusu et al. [11] introduced a Viewpoint Feature Histogram (VFH) descriptor that performs a 3D segmentation on the scene, calculates one single descriptor for the whole object surface and matches with model descriptors. Based on it, Clustered Viewpoint Feature Histogram (CVFH) [8] and Oriented, Unique and Repeatable Clustered Viewpoint Feature Histogram (OUR-CVFH) [12] were proposed. These methods could detect multiple objects with only depth information quickly but tend to fail if the segmentation could not localize the object instances on cluttered scenes. There are algorithms recognizing the objects by decomposing point clouds into geometric primitives [13,14,15], but these can not be applied to arbitrary organic objects. Iterative Closest Point (ICP) [9] is a method employed to minimize the difference between two clouds and always utilized to refine an initial pose. One of the promising approaches is the point pair feature algorithm proposed by Drost et al. [10] . The point pair features between every two model points are calculated and stored in a hash table. During matching, scene features are computed and matched with the model features using an efficient voting scheme. The algorithm does not need to undergo a 3D segmentation, is able to handle arbitrary organic objects and is utilized in many other works [16,17,18].

Many state-of-the-art algorithms [19,20,21,22,23,24] use RGB-D information in recognition. Hinterstoisser et al. [20] introduced multimodal-LINE (LINEMOD) to match scene templates with model templates using color gradient and normals. Based on LINEMOD, Hinterstoisse et al. [21] generated model templates with synthetic rendering of the object and performed pose verification with color and depth information. Gupta et al. [22] trained convolutional neural networks with semantically rich image and depth feature representation to detect objects. Brachmann et al. [23] built a random forest to obtain pixelwise dense predictions and, based on it, Krull et al. [24] used a convolutional neural network to learn to compare in the analysis-by-synthesis framework.

Algorithms using RGB-D usually perform better than those using depth only, since additional information is available. However, when RGB information is not available, which is true for some high resolution 3D sensors, or when the objects share a similar color, the algorithms can not present the best performance. Algorithms incorporating RGB may also be affected by illumination changes. By contrast, methods using depth information only will not be affected. In order to ensure the objects can be recognized under these circumstances, we focus on developing an algorithm based on the point pair feature approach [10]. A voting scheme on a reduced 2D search space is proposed and could work using sparse data. The disadvantage of this approach is that the computation time increases quickly with the scene point number since it computes and votes for the features between every two scene points. However, if most of the background points are removed, it can still present satisfactory performance.

In this paper, a point pair feature based pose estimation algorithm using depth information is proposed. To improve the efficiency of point pair feature approach, a boundary-based preprocessing method is proposed to remove background points and points belonging to foreground objects that are larger than the target. Then, the point pair feature approach [10] is performed on remaining points to obtain possible poses. For objects that are difficult to recognize from some viewpoints, an additional hash table is built and a model template selection method is proposed. A fast and accurate pose verification method considering both point correspondence and boundary correspondence is introduced to grade the poses and select the best pose. Our algorithm is proved to be able to compete with state-of-the-art algorithms using RGB-D information on published datasets.

The rest of the paper is organized as follows: Section 2 introduces our algorithm. Section 3 provides experiments to examine the algorithm and Section 4 gives the conclusion.

## 2. Method

**Figure 1:**
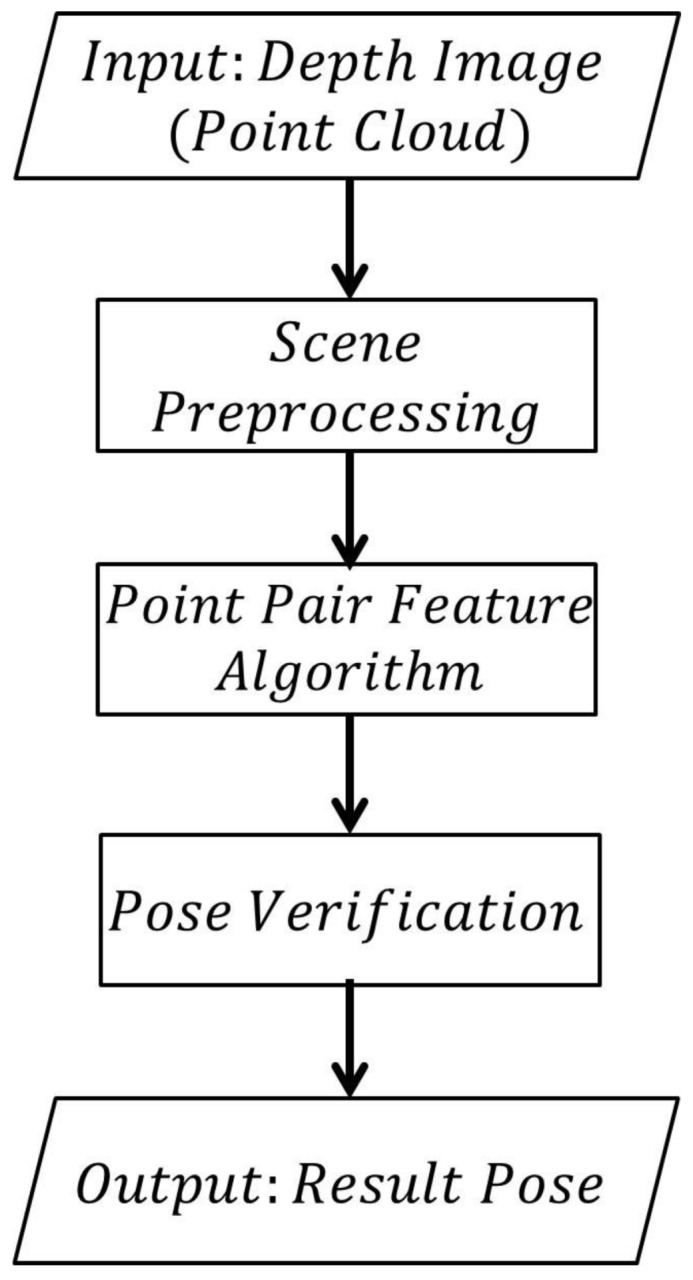
Pipeline of our algorithm.

In our algorithm, the model size of the target diam(M) is defined as the maximum 3D distance between every two points in the model. The pipeline of our estimation algorithm is presented in Figure 1. The input is a depth image or a point cloud. Firstly, scene preprocessing is performed to remove some irrelevant points. Then, a point pair feature algorithm is performed on the remaining points to generate pose candidates. These poses are evaluated by the pose verification method. The result poses are selected from poses with high scores.

### 2.1. Scene Preprocessing

**Figure 2:**
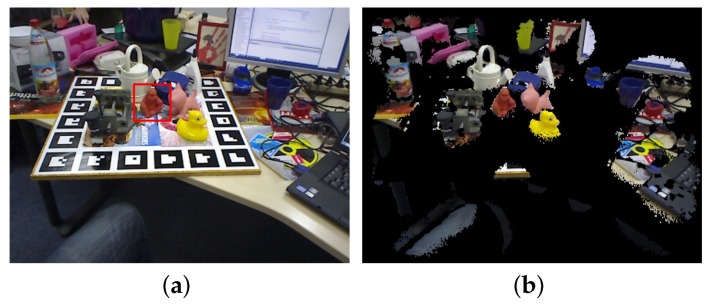
Illustration of scene preprocessing. (**a**) original image containing all scene points. The detection target is in the red bounding box; (**b**) the image after preprocessing. The black points are removed scene points while the color points are reserved points for matching.

Before matching, a boundary-based scene preprocessing is performed to remove the points belonging to background and foreground objects whose sizes are larger than diam(M), as shown in Figure 2.

For a depth image, the gradient of every pixel is calculated and, if the magnitude of a pixel is larger than a threshold (in our experiment, 10 mm), this pixel is considered as a boundary pixel. Then, based on the Connected-Component Labeling Algorithm of [25], the boundary pixels are clustered as curves if they meet the following conditions:

(1) Every pixel of a curve can find at least one pixel of the same curve among the eight surrounding pixels.
(2) The 3D distance between the corresponding 3D points of every two neighbor pixels is less than a threshold $d_{\mathrm{con}}$ (the threshold is slightly larger than the average point distance of the cloud).

**Figure 3:**
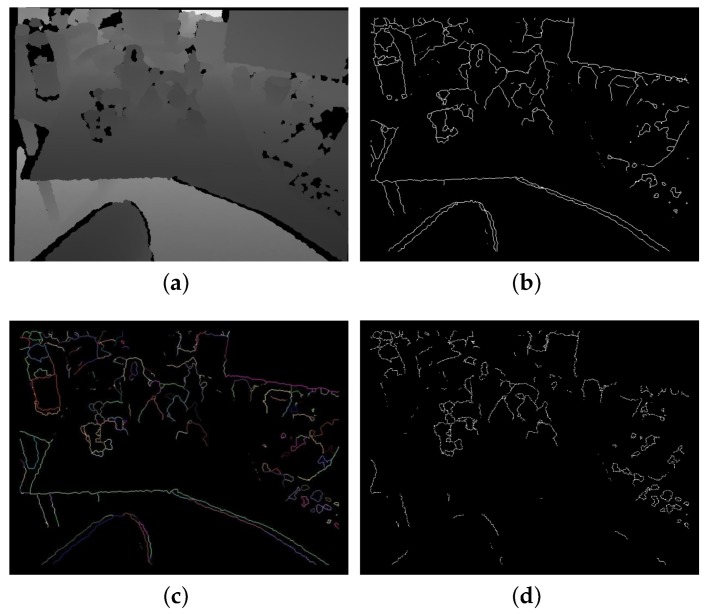
Example of boundary estimation and clustering (**a**) depth image; (**b**) estimated boundary pixels; (**c**) continuous boundary pixels are clustered into curves. The pixels belonging to the same curve have the same color; and (**d**) the boundary pixels after removing long curves.

The curves have two functions. Generally, the pixels of the same curve belong to the same object as long as $d_{\mathrm{con}}$ is not very large. If the length of a curve (the maximum 3D distance between every two points in the curve) is larger than diam(M), we assume that this curve does not belong to the target object and remove such curve consequently, as shown in Figure 3. Therefore, curves can be used to remove useless boundary pixels. The curves are also used in the boundary verification, which will be introduced in Section 2.4.3.

It should be noted that it is very difficult to ensure that all the boundary pixels of an object are in one curve, and, at the same time, the pixels of different objects are not connected. The former needs a large $d_{\mathrm{con}}$ that contradicts with the latter. Instead, we want to ensure that all the pixels in a curve belong to the same object, even if an object contains multiple curves. Therefore, $d_{\mathrm{con}}$ is set slightly larger than the average point distance.

**Figure 4:**
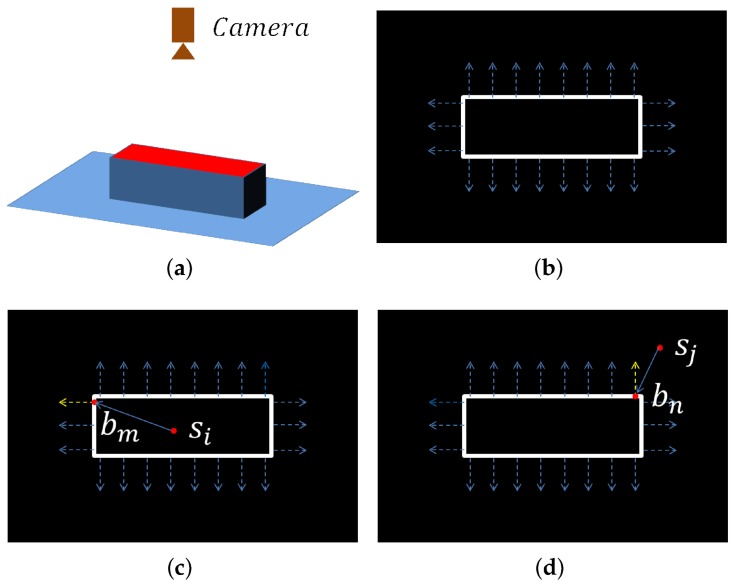
Illustration of how to distinguish foreground points with background points. (**a**) a cuboid on the plane and the camera is above the object; (**b**) the boundary points of the object (white points) and the gradient directions (blue dotted lines); (**c**) for a foreground point $s_{i}$ on the object and a boundary point $b_{m}$, the angle between the direction of $b_{m}$ (yellow dotted line) and the vector from $s_{i}$ to $b_{m}$ is less than 90°; (**d**) for a background point $s_{j}$ and boundary point $b_{n}$, the angle is larger than 90°. This difference is used to distinguish foreground points with background points.

Then, we introduce how to distinguish foreground points with background points using the boundaries. Suppose there is a cuboid on a plane and the camera is above it, as shown in Figure 4a. The boundary points and gradient directions are presented in Figure 4b. Consider a foreground point $s_{i}$ and a boundary point $b_{m}$ in Figure 4c. The angle between the gradient direction of $b_{m}$ and the vector from $s_{i}$ to $b_{m}$ is less than 90°. For a background point $s_{j}$ and a boundary point $b_{n}$ in Figure 4d, the angle between the gradient direction and the vector is larger than 90°. This difference is used to distinguish foreground points with background points.

Starting from a point $s_{i}$, the nearest boundary point $b_{m}$ in a direction is searched on the 2D boundary map. If the angle between the gradient direction of $b_{m}$ and the vector from $s_{i}$ to $b_{m}$ is less than 90° and the 3D distance between $s_{i}$ and $b_{m}$ is less than diam(M), $s_{i}$ is considered to find a valid intersection. This search is performed in 36 directions for $s_{i}$ (every 10° on the 2D map) and if the valid intersection number is larger than a threshold $N_{valid}$, $s_{i}$ is considered to be a foreground point and reserved. Otherwise, $s_{i}$ is removed. We found that the threshold 10∼20 is proper for most objects. The result of the process is shown in Figure 2.

### 2.2. Point Pair Feature

To obtain an initial guess of pose, we use the point pair feature algorithm [10]. Given an oriented scene point cloud or depth image and a target model, the point pair feature will be calculated for oriented points, respectively. By aligning the point locations and the normals of the point pairs sharing the same feature, the 6-DoF pose can be recovered. For two points $m_{1}$ and $m_{2}$ with normals $n_{1}$ and $n_{2}$, $d=m_{2}-m_{1}$, the feature is defined by Equation (1):

(1) $$F\left(m_{1},m_{2}\right)=(∥d∥,∠\left(n_{1},d\right),∠\left(n_{2},d\right),∠\left(n_{1},n_{2}\right)),$$

where $∠\left(a,b\right)\in \left[0;\pi \right]$ denotes the angle between two vectors. In the point pair, the first point $m_{1}$ is called the reference point and the second point $m_{2}$ is called the referred point.

During the offline stage, a hash table that stores all point pair features computed from the target model is built. The features are quantized and used as the key of hash table and the point pairs with the same feature are stored in the same slot.

**Figure 5:**
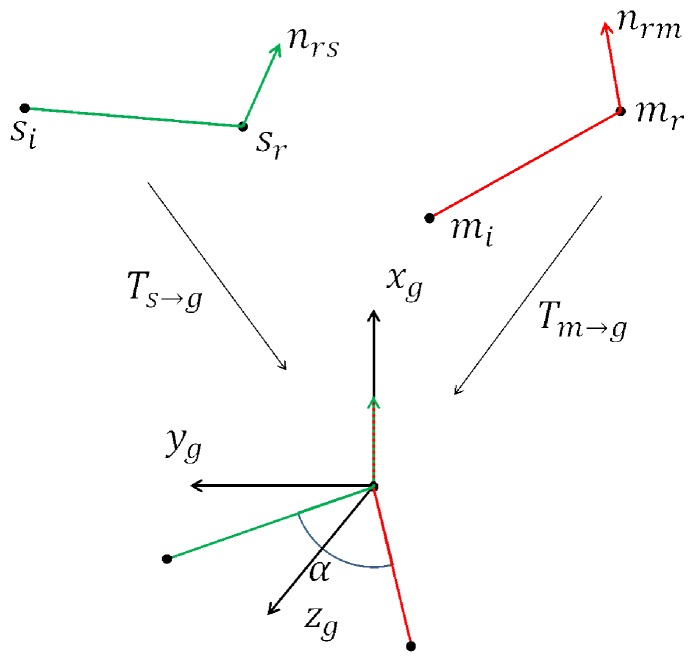
Transformation of corresponding points in model and scene. The transformation $T_{m\to g}$ translates the model point $m_{r}$ into the origin and rotates its normal $n_{r}^{m}$ onto the *x*-axis. $T_{s\to g}$ does the same for the scene point pair. In many cases, $s_{i}$ and $m_{i}$ will be misaligned, and the rotation $R_{x}\left(\alpha \right)$ around the *x*-axis with angle α is required to match them.

Given a depth image (scene cloud), pose hypotheses are computed by calculating the transformation between a scene point pair and a set of model point pairs. To make this search efficient, a voting scheme based on a 2D local coordinates is utilized. For the scene point pair ($s_{r},s_{i}$), suppose a corresponding point pair ($m_{r},m_{i}$) is found in the hash table *H*. Next, $s_{r}$ and $m_{r}$ are aligned in an intermediate coordinate system as shown in Figure 5. By rotating the model pair around the normal with an angle α, the referred points, $s_{i}$ and $m_{i}$ can be aligned. The 2D vector $\left(m_{r},\alpha \right)$ is defined as a local coordinate. The transformation is defined by Equation (2):

(2) $$s_{i}=T_{s\to g}^{-1}R_{x}\left(\alpha \right)T_{m\to g}m_{i},$$

and is explained in Figure 5.

In our task, only the reserved scene points from preprocessing are processed as reference points. For a reserved scene point $s_{r}$, point pairs with other scene points are computed and matched with model pairs using the above-mentioned process. Since the depth image is available, it is unnecessary to compute features with all other scene points. Instead, the referred scene points far from the reference point on the depth image are rejected to save time. A 2D accumulator is created to count the number of times every local coordinate is computed (vote). The top local coordinates (top five poses in our experiment) are selected based on their votes.

It should be noted that the removed points of scene preprocessing are still used as referred points since a few foreground points may also be removed.

Finally, the pose hypotheses are clustered such that all poses in one cluster do not differ in translation and rotation for more than a predefined threshold. Different from [10], who used the vote summation of clusters to select result pose, the average pose of every cluster is computed and stored along with the pose hypotheses for the verification because the accuracy of poses is improved by the pose clustering.

### 2.3. Partial Model Point Pair Feature

**Figure 6:**
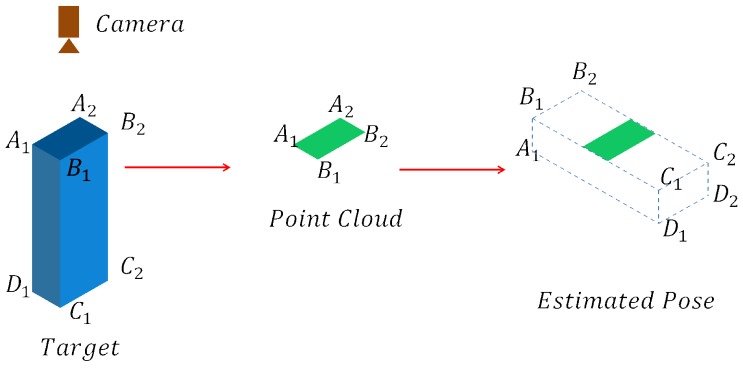
(**Left**) the camera is above the target cuboid; (**Middle**) only a small part of the target (plane $A_{1}B_{1}B_{2}A_{2}$) is taken; (**Right**) after voting with the hash table, the pose with the highest vote is wrong.

The hash table stores the features between every two points in the model to allow for the detection of any pose. However, if the camera views the target in such a viewpoint that only a small part of the target is visible, the point pair feature algorithm may fail to select the correct pose, as presented in Figure 6.

There are two reasons for this failure. One is that the features of the visible part are not distinguishable enough from other parts of the object. Another is that the normals of points near the boundaries in the scene could be quite different from those in the models. As a result, the correct poses can not get high votes in this case, which results in the detection failure.

Therefore, besides the hash table containing all model points, denoted as $H_{\mathrm{all}}$, additional tables $H_{\mathrm{add}}$ containing only a part of model points are built to handle these situations. For every scene point pair $\left(s_{r},s_{i}\right)$, the corresponding model point pairs are searched in all the tables and the top poses are selected from every table so that the correct poses are more likely to get high votes.

**Figure 7:**
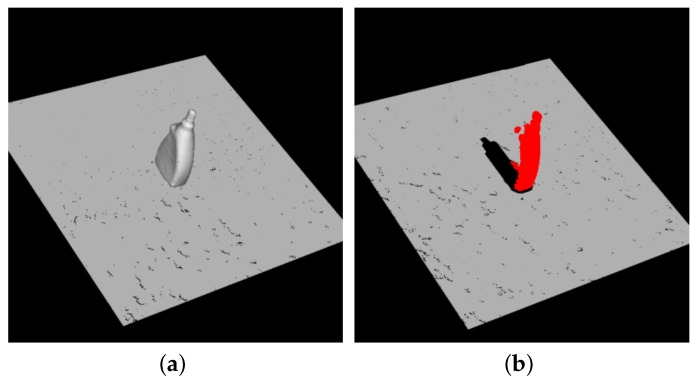
Generated clouds for template selection. (**a**) the synthetic scene of the object; (**b**) the partial cloud from a viewpoint. The red points belong to the object.

To select the model points for additional tables, the model *M* is viewed from viewpoints on the upper hemisphere and template clouds $MT=[MT_{1},MT_{2},\ldots ,MT_{n}]$ are generated. These templates are the candidates for $H_{\mathrm{add}}$. To select the best template, the following steps are performed:

(1) Create a synthetic scene of the object and generate partial clouds from thousands of viewpoints on the upper hemisphere, as presented in Figure 7.
(2) For every generated cloud SS, find the points belonging to the object and perform the point pair feature algorithm using these points as reference points with $H_{\mathrm{all}}$. Every reference point generates one pose. The score of SS is the number of points whose poses are correct. Find the nearest model template $MT_{j}$ based on the viewpoint of SS and pose of the object.
(3) The score of a template $MT_{j}$ is defined as the average score of the generated clouds whose nearest template is $MT_{j}$.
(4) After all clouds are processed, find the template with the lowest score. If the score is less than 50% of the average template score, this template is selected for the additional table.

In the template selection, the score of a template means the difficulty of recognizing the object in similar poses with $H_{\mathrm{all}}$. If the lowest score of the templates is much lower than the average score, it means that the object under similar poses is difficult to recognize with $H_{\mathrm{all}}$. Therefore, the additional hash table built with the template is necessary to handle these situations. Generally, no more than one template is selected to balance the trade-off between accuracy and computation time.

### 2.4. Pose Verification

**Figure 8:**
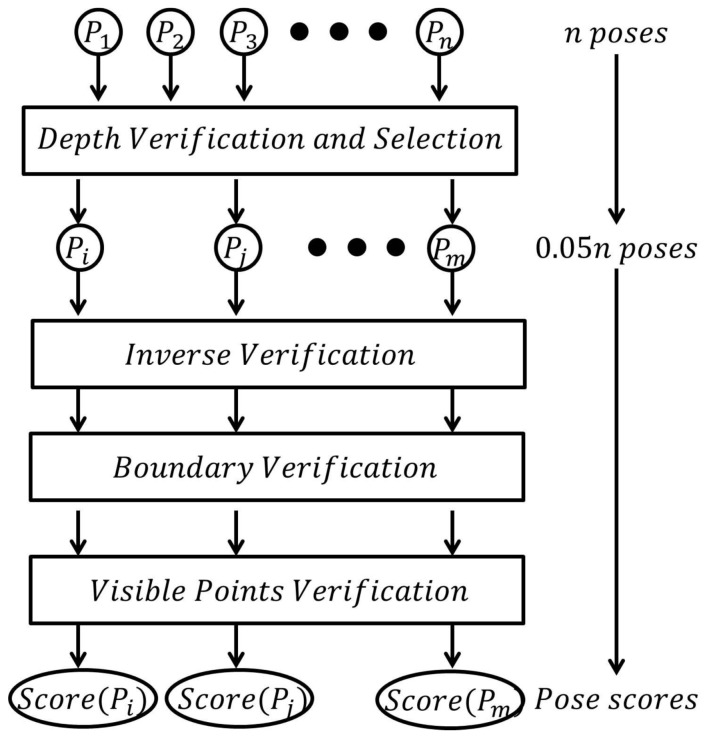
The pipeline of pose verification.

Different from [10], which uses the summation of votes of clusters to select result poses, we verify every pose proposed in the last step and select the best one. In order to improve the efficiency, and, at the same time, obtain a satisfying accuracy, the poses are firstly verified by the depth verification in Section 2.4.1. The top poses (in our experiment 5% poses) are selected and three scores are evaluated for them, which will be introduced in Section 2.4.2, Section 2.4.3 and Section 2.4.4, respectively, as presented in Figure 8. Finally, the pose with the highest score is selected as the result pose.

#### 2.4.1. Depth Verification

Given a pose $P_{i}$, the model points are transformed onto a depth map according to $P_{i}$. The score of $P_{i}$ in depth verification is the number of transformed model points whose depth difference from the pixel on the depth map is less than a threshold (in our experiment, the threshold is set as 0.02diam(M)). Depth verification is a fast, rough verification method and its function is to remove bad poses efficiently. The top poses are selected for next-stage verification.

#### 2.4.2. Inverse Verification

The inverse verification method is an improvement on the voxel-based verification method of [26] for wide space search. The idea of the verification is that, if the pose is correct, the transformed model points will find corresponding scene points near them. [26] divided the scene space into small voxels and every voxel stores the scene point within it. It built another hash table to access the voxels with a 3D coordinate efficiently. To verify a pose $P_{i}$, [26] transformed all model points into scene space and checked whether there are scene points near the transformed model points by the voxel hash table.

However, this is difficult to implement when the scene space is very wide. If the length, width and height of the scene space are 1000 mm and the voxel length is 1 mm, $1000^{3}$ voxels are necessary to cover the scene space. The storage and time for it are unacceptable. Therefore, instead of transforming the model into scene space, we do it inversely:

(1) During the offline stage, divide the model space into small voxels and each voxel stores the model point in it.
(2) Build a hash table to efficiently access the voxels with 3D coordinates.
(3) To verify a pose $P_{i}$, transform the model center $c_{m}$ into scene space according to $P_{i}$: $c_{\mathrm{mt}}=P_{i}c_{m}$. Select scene points from the depth image whose distance from $c_{\mathrm{mt}}$ is less than 0.5diam(M).
(4) Transform the selected scene points into model space by $P_{i}^{-1}$. For every transformed scene point $st_{j}$, if the voxel $st_{j}$ contains a model point, it means that $st_{j}$ has a corresponding model point. The inverse score of $P_{i}$, which is denoted as $S_{\mathrm{inverse}}\left(P_{i}\right)$, is the number of transformed scene points with corresponding model points.

The advantage of inverse verification is threefold:

(1) It saves time and storage to build a voxel map for a model instead of a scene.
(2) By using the model voxel map, it is quick to search corresponding model points for transformed scene points.
(3) By transforming only the scene points around the transformed model center $c_{\mathrm{mt}}$, the efficiency is improved.

**Figure 9:**
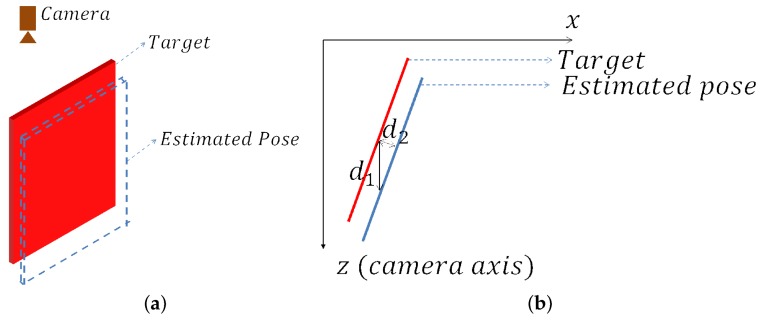
The difference between depth verification and inverse verification. (**a**) the target is the red planar object and the blue dotted lines present the estimated pose; (**b**) the object and pose shown in 2D. Suppose the *z*-axis is the camera axis, the red line is the scene points of the object we want to estimate and the blue line is the estimated pose. If the pose is evaluated by the inverse verification method, the error should be $d_{2}$. However, if it is evaluated by the depth method, the error will be $d_{1}$, which is much larger.

Then, the question may come that since depth verification can do the same work, why is inverse verification used? It is true that depth verification is faster and can also calculate the distance between model and scene points. However, the accuracy of depth verification is worse than inverse verification. Suppose the target is a planer object, as shown in Figure 9. The transformation error between the estimated pose and ground truth is approximately equal to $d_{2}$. However, if the pose is evaluated by depth verification, the average distance error will be $d_{1}$, which is much larger than $d_{2}$. Therefore, the depth verification method is not accurate when the depth gradient is large.

#### 2.4.3. Boundary Verification

Different from the inverse verification that evaluates poses in 3D model space, the boundary verification is performed in a 2D image because verification in 3D costs too much time and storage. A scene boundary map $B_{scene}$ is computed from the depth gradient, as introduced in Section 2.1. The model boundary map for pose $P_{i}$, denoted as $B_{model}\left(P_{i}\right)$, is obtained by transforming model points to scene space according to $P_{i}$, projecting the points onto the plane perpendicular to the camera axis and extracting the contour of the projected image.

Given $B_{scene}$ and $B_{model}\left(P_{i}\right)$, if two pixels in the same position (row and column) of the two images are both boundary pixels, these two pixels are called corresponding boundary pixels and the model boundary pixel is called a fitted pixel. If many boundary pixels of $B_{model}\left(P_{i}\right)$ are fitted pixels, it means the model boundary matches well with scene boundary in 2D and the boundary score of $P_{i}$ should be high.

**Figure 10:**
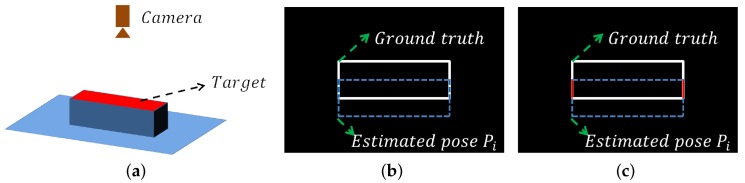
An example of boundary verification considering curves. (**a**) the cuboid target is on the plane and the camera is above them; (**b**) the white pixels are $B_{scene}$ and also present the ground truth of the object. All of the white pixels belong to the same curve. An estimated pose $P_{i}$ is presented by the blue dot line ($B_{model}\left(P_{i}\right)$); (**c**) since only a small part of the curve (red pixels) matches the $B_{model}\left(P_{i}\right)$, this curve is invalid for $P_{i}$.

In Section 2.1, scene boundary pixels are clustered into curves by their continuity and this clustering information is utilized in boundary verification. In general, boundary pixels from the same curve belong to the same object. If only a small part of pixels of a curve correspond to the pixels of $B_{model}\left(P_{i}\right)$, these corresponding boundary pixels are considered to be invalid for $P_{i}$, as presented in Figure 10.

Therefore, the boundary verification is performed by the following steps:

(1) Spread the boundary pixels in $B_{scene}$ among neighboring pixels to allow for small pose error.
(2) Given a pose $P_{i}$, for every boundary pixel in $B_{model}\left(P_{i}\right)$, if it is a fitted pixel, record the curve that the corresponding scene boundary pixel belongs to.
(3) For a curve, if a certain percentage $R_{curve}$ of its pixels correspond to $B_{model}\left(P_{i}\right)$, this curve is considered to be valid for $P_{i}$.
(4) Search corresponding boundary pixels for $B_{model}\left(P_{i}\right)$ again. This time, only scene boundary pixels of curves valid for $P_{i}$ are searched. The boundary score of $P_{i}$ is the number of fitted pixels divided by the number of boundary pixels in $B_{model}\left(P_{i}\right)$:
  (3) $$S_{\mathrm{boundary}}\left(P_{i}\right)=\frac{number\;of\;fitted\;pixels}{number\;of\;boundary\;pixels\;in\;B_{model}\left(P_{i}\right)}.$$

#### 2.4.4. Visible Points Verification

The inverse verification counts the number of scene points with corresponding model points. The more points are matched, the higher the score is. However, the visible point number of the object may be small in some poses, for example, the cuboid in Figure 6. In this case, the correct pose will get a low score and cause recognition failure. Therefore, the visible score $S_{\mathrm{visible}}\left(P_{i}\right)$ is computed to make up for it by the following steps:

(1) Compute the visible model point based on $P_{i}$ and camera viewpoint.
(2) Transform the visible points onto depth image according to $P_{i}$. Similar to the depth verification, count the number of fitted pixels whose depth difference from estimated depth is less than a threshold.
(3) $S_{\mathrm{visible}}\left(P_{i}\right)$ is defined as the fitted pixel number divided by the visible point number.

#### 2.4.5. Select Result Pose

The score of a pose is the product of the three scores:

(4) $$Score\left(P_{i}\right)=S_{\mathrm{inverse}}\left(P_{i}\right)S_{\mathrm{boundary}}\left(P_{i}\right)S_{\mathrm{visible}}\left(P_{i}\right).$$

If only one instance is detected in the scene, ICP refinement [9] is performed on top poses (in our experiment, top 10 poses) and the pose after refinement with highest verification score is selected as the result pose. In case of selecting multiple poses, we firstly select the rough result poses and then perform ICP on them.

## 3. Experiment

We compare our algorithm with state-of-the-art algorithms on the ACCV dataset of [21] and on the Tejani dataset of [19]. We implemented our algorithm in C++ on an Intel Core i7-7820HQ CPU with 2.90 GHz and 32 GB RAM. Multicore enhancement like GPU was not used.

In our experiments, the model clouds and scene clouds were subsampled so that the model point numbers were around 500. Some parameters are presented in Appendix A. For a 3D model *M*, having the ground truth rotation *R* and translation T and the estimated rotation $\bar{R}$ and translation $\bar{T}$, we use the equation of [21] to compute the matching score of a pose:

(5) $$m=\overset{avg}{\underset{x\in M}{}}||\left(Rx+T\right)-\left(\bar{R}x+\bar{T}\right)\left|\right|.$$

The pose is thought to be correct if $k_{m}diam\left(M\right)\geq m$. Following state-of-the-art algorithms, we set $k_{m}=0.1$ as the threshold in our experiments. For ambiguous objects, Equation (6) is used:

(6) $$m=\overset{avg}{\underset{x_{1}\in M}{}}\overset{min}{\underset{x_{2}\in M}{}}\left|\right|\left(Rx_{1}+T\right)-\left(\bar{R}x_{2}+\bar{T}\right)\left|\right|.$$

### 3.1. ACCV Dataset

This dataset consists of 15 objects and there are over 1100 images for every object. We skipped two objects, the bowl and the cup since state-of-the-art algorithms removed them.

**Figure 11:**
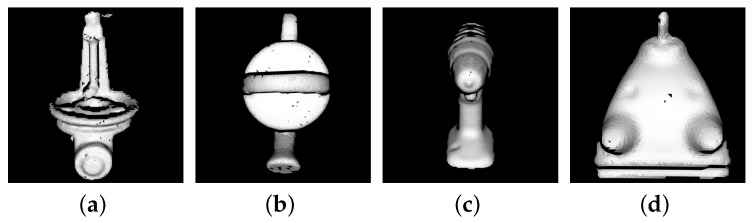
Selected template of (**a**) Benchvise; (**b**) Can; (**c**) Driller; (**d**) Iron.

**Figure 12:**
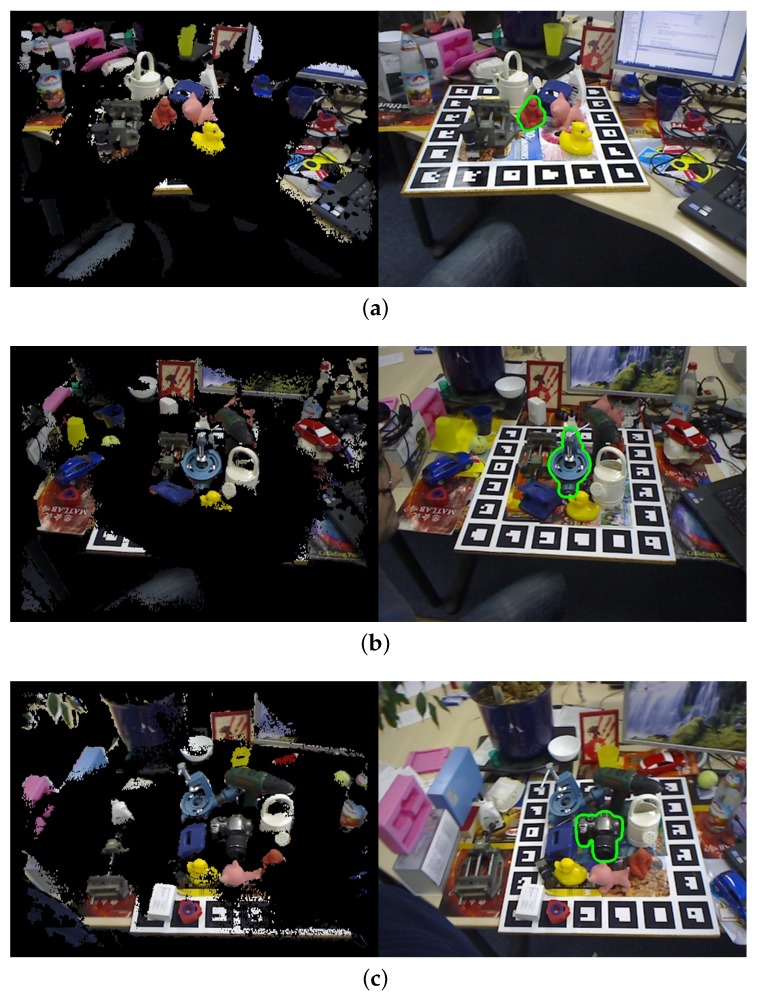
Our detection on (**a**) Ape; (**b**) Benchvise; (**c**) camera of ACCV dataset; (**Left**) image after scene preprocessing. (**Right**) detection result. The green points show the detection result.

Model templates were selected for nine models: Benchvise, Can, Cat, Driller, Glue, Hole puncher, Iron, Lamp, Phone and some of them are presented in Figure 11. The performance of the algorithms are in Table 1 and some detection results are shown in Figure 12.

Same as [10,17], we only used depth information in the experiment, but we achieved the highest accuracy for seven objects and highest average accuracy.

### 3.2. Tejani Dataset

**Figure 13:**
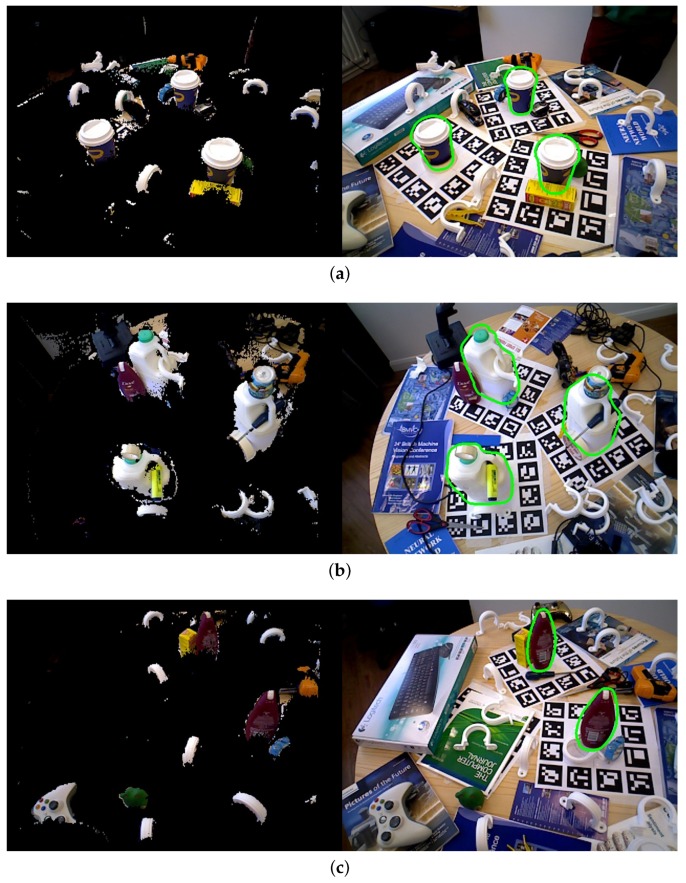
Our detection on (**a**) coffee (**b**) milk; (**c**) shampoo of Tejani dataset. (**Left**) image after scene preprocessing; (**Right**) detection result. The green points show the detection results.

The Tejani dataset [19] contains six objects with over 2000 images and there are two or three instances in every image with their ground truth poses. Model templates were selected for four models: Camera, Juice, Milk and Shampoo. Following [19,28], we reported the F1-score of the algorithms in Table 2. Some detection results are shown in Figure 13.

The results of the compared algorithms come from [28]. The LINEMOD [20] and LC-HF [19] used RGB-D information, Kehl [28] used RGB only and our algorithm used depth information only. Our algorithm presented a better average accuracy than the compared algorithms.

### 3.3. Computation Time

**Figure 14:**
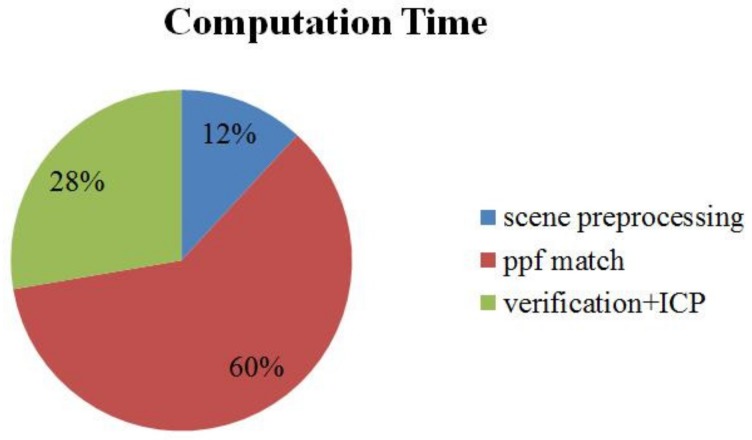
Components of the computation time of our algorithm.

The components of our computation time are presented in Figure 14. The average computation time for the ACCV datasets, including the time for the additional hash table, for one scene was 1018 ms times faster than the point pair feature algorithm of [10], whose computation time was reported to be 6.3 s [21], thanks to the scene preprocessing. Using an additional hash table for model template increased the computation time by 106 ms.

In our algorithm, the most important algorithm is the subsampling size and we explore how it affects the performance of the algorithm on ACCV dataset. For every object, the model and scene clouds were subsampled such that the model point number $N_{model}$ was around 300, 500, 700 and 900. The recognition rate and computation time are presented in Table 3. From $N_{model}=300$ to $N_{model}=500$, the recognition rate increases by 6.7% and computation time increases by 530 ms. From $N_{model}=500$ to $N_{model}=900$, the recognition rate only increases by 0.8%, but the computation time increases by 2558 ms. Therefore, $N_{model}=500$ was selected in our experiments.

### 3.4. Contribution of Each Step

In order to explore the contribution of the scene preprocessing, additional hash table and pose verification, we conducted experiments on the ACCV dataset. In the first experiment, only the scene preprocessing was not performed for all 13 of the objects. In the second experiment, only the verification was not performed and the poses were selected by the simple depth verification. In the third experiment, only the additional hash table was not performed for the eight objects. The results of the first and second experiment are presented in Table 4 and the result of the third experiment is presented in Table 5. We can see from the tables that, with the scene preprocessing, the computation time decreases by 79.4% with only 0.3% decrease in recognition rate. The pose verification improves the recognition rate by 29.8% and the additional hash table improves that of the 8 objects from 96.1% to 97.5%.

## 4. Conclusions

This paper proposed an object recognition and pose estimation algorithm using depth information. The scene preprocessing method could improve the efficiency in cluttered scenes with boundary pixels. Model template selection method and the pose verification method improved the accuracy from 78.9% to 97.8% on the ACCV dataset. Our algorithm outperformed state-of-the-art algorithms, including algorithms using both color information and depth information, on a large number of scenes.

## Figures and Tables

**Table 1:** Accuracy of the algorithms on the ACCV dataset of [21].

Algorithm	Proposed	Hinterstoisser [17]	Drost [10]	DTT [27]	LINEMOD [20]
Ape	97.2%	**98.5%**	86.5%	95.0%	86.3%
Bench V.	98.5%	**99.8%**	70.7%	98.9%	98.0%
Cam	**99.6%**	99.3%	78.6%	98.2%	93.4%
Can	**99.2%**	98.7%	80.2%	96.3%	91.3%
Cat	98.5%	**99.9%**	85.4%	99.1%	97.9%
Driller	**98.6%**	93.4%	87.3%	94.3%	91.8%
Duck	96.3%	**98.2%**	46.0%	94.2%	91.4%
Eggbox	**99.9%**	98.8%	97.0%	99.8%	99.8%
Glue	90.7%	75.4%	57.2%	**96.3%**	80.9%
Hole P.	**98.3%**	98.1%	77.4%	97.5%	90.5%
Iron	**98.9%**	98.3%	84.9%	98.4%	95.5%
Lamp	96.2%	96.0%	93.3%	**97.9%**	97.5%
Phone	**98.9%**	98.6%	80.7%	88.3%	88.3%
Average	**97.8%**	96.4%	78.9%	96.5%	92.5%

**Table 2:** F1-score of the algorithms on the Tejani dataset of [19].

Algorithm	Proposed	Kehl [28]	LC-HF [19]	LINEMOD [20]
Camera	0.603	**0.741**	0.394	0.589
Coffee	**0.991**	0.983	0.891	0.942
Joystick	0.937	**0.997**	0.549	0.846
Juice	**0.977**	0.919	0.883	0.595
Milk	**0.954**	0.780	0.397	0.558
Shampoo	**0.999**	0.892	0.792	0.922
Average	**0.910**	0.885	0.651	0.740

**Table 3:** Recognition rate and computation time (ms/scene) against model point number on the ACCV dataset.

Model Point Number	300	500	700	900
Recognition rate	91.1%	97.8%	98.2%	98.6%
Computation time	488	1018	1923	3576

**Table 4:** Contribution of scene preprocessing and pose verification on all 13 of the objects.

Algorithm	Proposed	No Preprocessing	No Verification
Recognition rate	97.8%	98.1%	68.0%
Computation time	1018	4951	736

**Table 5:** Contribution of additional hash table on 8 objects.

Algorithm	Proposed	No Additional Hash Table
Recognition rate	97.5%	96.1%
Computation time	1009	903

## Appendix A

For different objects, the valid intersection number threshold $N_{valid}$ for scene preprocessing and the curve corresponding percentage threshold $R_{curve}$ for boundary verification were different in the experiment. They are provided in this section (Table A1 for the ACCV dataset and Table A2 for the Tejani dataset), in order to allow exact re-implementation.

**Table A1:** Parameters of the ACCV dataset.

Object	$N_{\mathrm{valid}}$	$R_{\mathrm{curve}}$
Ape	10	0.7
Bench V.	16	0.3
Cam	16	0.7
Can	14	0.5
Cat	18	0.5
Driller	14	0.5
Duck	10	0.5
Eggbox	14	0.5
Glue	20	0.7
Hole P.	14	0.3
Iron	14	0.3
Lamp	16	0.3
Phone	18	0.7

**Table A2:** Parameters of the Tejani dataset.

Object	$N_{\mathrm{valid}}$	$R_{\mathrm{curve}}$
Camera	10	0.7
Coffee	16	0.7
Joystick	10	0.7
Juice	18	0.7
Milk	16	0.7
Shampoo	20	0.7
